# Investigating developmental changes in sensory processing: visual mismatch response in healthy children

**DOI:** 10.3389/fnhum.2013.00922

**Published:** 2013-12-30

**Authors:** Katherine M. Cleary, Franc C. L. Donkers, Anna M. Evans, Aysenil Belger

**Affiliations:** ^1^Department of Psychiatry, School of Medicine, University of North Carolina at Chapel HillChapel Hill, NC, USA; ^2^Department of Psychology, Tilburg UniversityTilburg, Netherlands

**Keywords:** mismatch negativity, visual mismatch response, vMMN, children, developmental psychology, spatial frequency processing, ERP, EEG

## Abstract

The ability to detect small changes in one's visual environment is important for effective adaptation to and interaction with a wide variety of external stimuli. Much research has studied the auditory mismatch negativity (MMN), or the brain's automatic response to rare changes in a series of repetitive auditory stimuli. But recent studies indicate that a visual homolog to this component of the event-related potential (ERP) can also be measured. While most visual mismatch response (vMMR) studies have focused on adult populations, few studies have investigated this response in healthy children, and little is known about the developmental nature of this phenomenon. We recorded EEG data in 22 healthy children (ages 8–12) and 20 healthy adults (ages 18–42). Participants were presented with two types of task irrelevant background images of black and gray gratings while performing a visual target detection task. Spatial frequency of the background gratings was varied with 85% of the gratings being of high spatial frequency (HSF; i.e., standard background stimulus) and 15% of the images being of low spatial frequency (LSF; i.e., deviant background stimulus). Results in the adult group showed a robust mismatch response to deviant (non-target) background stimuli at around 150 ms post-stimulus at occipital electrode locations. In the children, two negativities around 150 and 230 ms post-stimulus at occipital electrode locations and a positivity around 250 ms post-stimulus at fronto-central electrode locations were observed. In addition, larger amplitudes of P1 and longer latencies of P1 and N1 to deviant background stimuli were observed in children vs. adults. These results suggest that processing of deviant stimuli presented outside the focus of attention in 8–12-year-old children differs from those in adults, and are in agreement with previous research. They also suggest that the vMMR may change across the lifespan in accordance with other components of the visual ERP.

## Introduction

The human brain is constantly responding to changes in sensory stimuli, even if these changes do not pass into conscious awareness. Mismatch negativity (MMN), or the brain's response to infrequent changes in a series of repetitive stimuli (Näätänen and Escera, 2000), is an element of the Event-Related Potential (ERP) that allows for the investigation of the neural correlates of (automatic) change detection in the environment. MMN is typically measured when the subject's attention is directed away from the stimulus, and manifests as a difference wave computed by subtracting the ERP to a frequently-occurring standard stimulus from the ERP to a rarely-occurring deviant stimulus. The MMN can be measured relatively early in development and is generally viewed as the outcome of a mechanism that compares the current sensory input to memory traces formed by previous repetitive inputs, and signals a mismatch between them (e.g., Naätänen et al., 2005, 2007).

MMN has mainly been investigated in the auditory modality, but recent studies have characterized this difference wave in the visual modality as well (see Pazo-Alvarez et al., 2003; Czigler, 2007, for reviews). Recent research (reviewed by Pazo-Alvarez et al., 2003 and Czigler, 2007) has provided convincing evidence that the brain can unconsciously detect small changes in visual environment. Visual MMN (vMMN) is an occipital-parietal negativity computed by subtracting the ERP to a frequently-occurring standard stimulus from the ERP to a rarely-occurring deviant stimulus in the visual modality. vMMN usually occurs around 100–250 ms post-stimulus presentation and to date has been primarily studied in typically-developing adults. Visual MNN has been observed in response to unattended changes in color (Czigler et al., 2002, 2006; Berti, 2009), line orientation (Kimura et al., 2009; Czigler and Sulykos, 2010), stimulus position in the visual field (Berti, 2009; Muller et al., 2012), emotional faces (Chang et al., 2010; Gayle et al., 2012; Stefanics et al., 2012), and spatial frequency (Fu et al., 2003; Heslenfeld, 2003).

Like the more frequently studied auditory MMN, differences in the specific paradigms employed and, in some cases, differences in populations studied, may yield different patterns of vMMN. In early vMMN studies, there has been some debate as to whether this negativity represents the refractory effect of the visual stimulus itself or a true detection of change based on building up of a memory trace for the repeated stimulus and a “comparison” of the deviant stimulus features against this trace. Kimura et al. (2009) addressed this question by presenting healthy subjects with two paradigms, the equiprobable (all types of stimuli presented at equal frequencies) and the oddball (standard stimuli 80% of presentations and deviant stimuli 20%). In the equiprobable paradigm, bar stimuli in five different types of orientations were presented; a control bar stimulus was presented twenty percent of the time, equally as likely to be viewed as any of the other four orientations. In the equiprobable paradigm change-specific neuronal populations should not be activated. In the oddball sequence, two bar stimuli with the two closest line orientations were presented: the deviant stimulus twenty percent of the time, and the standard stimulus eighty percent of the time. The authors compared deviant/standard, deviant/control, and control/standard pairings, and found two negativities when comparing deviant stimuli to standard stimuli; one at 100–150 ms and another at 200–250 ms. However, when they compared deviant stimuli to control stimuli, only the later negativity was elicited. The authors concluded that the early negativity is related to the refractory effect while the later one is related to the memory component of stimulus change detection. Similarly, Czigler et al. (2006) found two occipital/centro-parietal negativities in healthy adults viewing a set order of color grids that was periodically displaced. One negativity occurred at 100–140 ms post-stimulus and another at 210–280 ms post-stimulus. The purpose of the set pattern of alternating colors was to determine if the vMMN was related to change in stimuli themselves or a detection of deviance from a pre-established pattern of change in stimuli. Only the second later negativity at 210–280 ms was elicited when the pattern of color grids was violated, indicating that this later waveform reflects comparison to an established memory trace for the stimulus pattern and not stimulus change *per se*. These findings indicate that, in the visual modality, change detection may involve a 2-step process: a first “sensory” change detection, occurring earlier, and possibly processed at a more “local” level in primary sensory cortices; and a second, occurring slightly later, and possibly depending upon the contrasting of the current stimulus with an established “contextual memory trace” through interactions between visual sensory and higher order associated cortical regions.

Despite a growing number of visual mismatch response (vMMR) studies in adults, there is comparatively little research on the vMMR in children. A recent study by Clery et al. (2012) used dynamic deformations in a circle slowly becoming an ellipse to examine vMMR in healthy adults, as well as in healthy children ages 8–14. While in adults the vMMR was observed as an occipital-parietal negativity occurring around 210 ms post-stimulus, in children, three successive negativities originating over fronto-central electrode positions were observed between 150 and 330 ms. In addition, a larger late mismatch *positive* response was observed in children around 450 ms post-stimulus. The authors conclude that not only is the vMMR immature in children up to 14 years of age, but the successive negative potentials may reflect a sequential visual processing of deviancy that is not present in the mature brain. Processing of visual deviancy during development may require several distinct steps that are not necessary for adults, and may be related to immature selective attention processes or underdeveloped connectivity across cortical regions. Scalp topography maps suggested equal temporal recruitment of the dorsal and ventral pathways in adults, but the involvement of right parietal areas in the late positive potential observed in children may indicate that the dorsal pathway is engaged later in stimulus change detection processing in children. It is worth noting, however that the stimuli used in the Clery et al. study featured changes in both form and motion, and the authors hypothesize that these two stimulus properties may be processed separately in children, with maturation of the visual system leading to better integration of multiple stimulus properties. Currently no studies have investigated the vMMR in children treating changes in stimulus form and motion as separate deviant events. Studies using static stimuli that probe changes in physical form or dynamic stimuli with constant physical properties would help confirm this theory. Also worth noting is that the age range investigated in the Clery et al. study comprised a good portion of late childhood and adolescence. Since many important neurophysiological changes occur during adolescence, vMMR may be different in the younger portion of their sample compared to the older portion of their participants. The authors also note that developmental changes in vMMR appear more drastic than those in the auditory modality. Other studies have also reported latency decreases in vMMR with age up to approximately age 16 (Tomio et al., 2012). This latency difference may indicate improved cognitive processing until the late teenage years, possibly associated with improved connectivity resulting from brain maturation. In particular, Tomio et al. conclude that increasing age affords increasing ability to discriminate stimulus properties pre-attentively, and hypothesize that difficulty of stimulus property discrimination may affect latency differences. These differences are seen in other studies that have investigated vMMR across development using different stimuli, such as color differences (Horimoto et al., 2002), which appear to be developmentally mature at 7–13 years of age and can even be observed in mentally retarded (MR) children. Therefore, color modality may be easier to discriminate than the black and white stimulus pattern used by Tomio et al., and may require less advanced stimulus discrimination ability.

While a small number of recent studies, described above, have investigated vMMR in children, specific differences in the mismatch response at various stages in development and across different paradigms are still unclear. In addition, understanding of the neurobiology of developmental differences in vMMR is still in its infancy. In the current study, we aim to further characterize the vMMR in a sample of 8–12-year-old children. This age range is comparable to the age range used in the Clery et al. (2012) study but we chose to limit the upper age range to twelve in order to examine a slightly narrower defined age group. We compared the vMMR to deviant task-irrelevant background stimuli in children to the vMMR of adults while both groups were occupied performing a simple target detection task. We hypothesized that a vMMR would be observed to changes in background stimuli in both groups. Because our stimuli deviated only in form, rather than in form and motion as in a previous study with children in this age range (Clery et al., 2012), we hypothesized the appearance of one negative occipital deflection in the difference wave for both groups.

## Materials and methods

### Participants

We collected EEG data from 20 healthy adults between the ages of 18 and 42 [mean age = 26.6, (*SD* = 5.65); 10 females; 78% right-handed] and 22 healthy children between the ages of 8 and 12 [mean age = 10.4, (*SD* = 1.43); 13 females; 85% right-handed]. All participants reported no current, past, or family history of substance abuse, no neurological/neuropsychiatric disorders, no seizure disorder with evidence of seizure activity within the past 12 months, no significant physical impairments or limitations, no history of head trauma or loss of consciousness, and were not currently taking any antipsychotic medications. Participants reported normal or corrected-to-normal vision. One child was excluded from further analysis due to excessive sleepiness during recording, resulting in noisy data.

Participants were recruited from multiple venues, including a university-based mass email system and local community and parent groups. Participants received $30 for taking part in the study and a certificate with a graphical image of their brain waves to take home. Adult participants gave informed consent, and minor participants provided written assent while their parents provided parental permission as approved by the University of North Carolina Institutional Review Board.

### Experimental procedure

**Figure 1:**
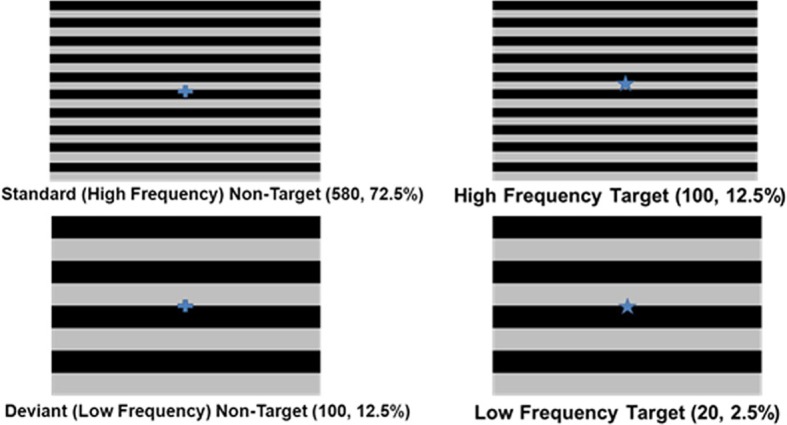
**Task stimuli and design**.

Visual MMN Paradigm: Continuous EEG data was recorded while participants were presented with target (15% probability) and non-target images (85% probability) displayed at fixation in front of two types of task irrelevant background images of gratings. The target (2 × 2 cm) was a blue star presented in the center of a black and gray grating, while the non-target was a blue crosshair (2 × 2 cm) in the same location (visual angle of star and crosshair <2 × 2°). Four different stimulus conditions were created (see Figure 1): high spatial frequency (HSF) background with target image placed in center (12.5%), low spatial frequency (LSF) background with target image placed in center (2.5%), HSF background with non-target image placed in the center (72.5%) and LSF background with non-target image place in the center (12.5%). All images were 960 × 720 pixels and consisted of gray and black bars in a repeating pattern. LSF images consisted of four cycles of gray and black bars while HSF images consisted of 10 cycles. LSF images (15% probability) served as deviant background stimuli while HSF images (85% probability) served as standard background stimuli. Our primary events of interest were the standard non-target HSF images with a blue crosshair in the center (HFNT), and the deviant non-target LSF images with a blue crosshair in the center (LFNT). Participants were told that they would view a series of pictures and that their task was to ignore the background gratings and press a button each time an image of a star appeared at the center of the screen. Target events were omitted from analysis and were only included in the experiment in order to make sure that participant paid attention to the screen. No training blocks were provided. Stimuli were presented in a pseudorandom order (i.e., no deviant non-target stimulus was followed by another deviant non-target stimulus). Five runs of 5 min each were presented, with 160 images per run and 800 images total. The total session (including electrode preparation, breaks, and cleanup) lasted no more than 90 min. Images were presented for 750 ms duration, with an interstimulus interval of 1000 ms (offset to onset).

### Electrophysiological recording

Participants were seated comfortably in a sound-attenuated, dimly lit booth and were instructed to avoid excessive movement, tension of facial muscles, horizontal eye movements, or speaking. Images were displayed on a 19-inch Dell flat panel monitor with a 60 Hz refresh rate. Participants were seated 100 cm away from the stimulus monitor adjusted to be at eye level. Stimulus presentation was controlled by CIGAL software, version 17.2 (Voyvodic, 1999). Continuous EEG data were collected using an elastic cap containing 18 electrodes, with only 13 electrodes used to collect data: at frontal (F3, Fz, F4), central (T7, C3, Cz, C4, T8), parietal (P3, Pz, P4), and occipital (O1, O2) scalp locations. The right mastoid served as the reference electrode and AFz as the ground. Bipolar recordings of the vertical and horizontal electro-oculogram (EOG) were obtained by electrodes placed above and below the right eye and on the outer canthus of each eye, respectively. EEG and EOG data were sampled at a rate of 500 Hz and bandpass filtered online between 0.05 and 100 Hz, with a narrow 60 Hz notch filter used to reduce main power frequency interference. Continuous data were analyzed off-line using NeuroScan 4.4 software (Neurosoft, Inc., Sterling, VA, USA).

### Data processing

Response latencies and percentage of correct responses to target stimuli were calculated for each subject. All incorrect trials or trials containing responses less than 200 ms and greater than 1000 ms from onset of the target were excluded from further analyses. Continuous EEG data was filtered offline with a 30 Hz (24 dB/octave) zero phase shift Butterworth low-pass filter and visually inspected for movement artifacts. EEG data sets from each participant were corrected for eye-movements using regression analysis as implemented in Neuroscan Edit 4.4 (Semlitsch et al., 1986). Continuous EEG data from all channels were epoched using a 200 ms pre-stimulus baseline period and a 1000 ms post-stimulus period. Individual epochs were passed through an automatic artifact detection algorithm to remove epochs with EEG activity in excess of –100 μV or +100 μV. After pre-processing, the number of remaining trials for the main stimulus conditions of interest were as follows, standard non-target: 534.05 (range 439–565) for adults vs. 427.48 (range 209–558) for children [*F*_(1, 39)_ = 19.58, *p* < 0.001]; deviant non-target: 93.65 (range 83–100) for adults vs. 74.81 (range 34–97) for children [*F*_(1, 39)_ = 21.42, *p* < 0.001]. ERPs were obtained by averaging the baseline corrected EEG epochs for each stimulus category and for each participant.

The P1 and N1 were identified by an automatic peak detection procedure, defined as the most positive and negative peak (as appropriate) within a specified window after stimulus onset. For P1 and N1, peak windows were determined based on the relevant peak in a visual inspection of grand averages at electrode positions O1, O2, F3, Fz, F4, C3, Cz, and C4. For the occipital channels, P1 peak detection windows for children and adults were defined as 80–150 ms for standard non-target stimuli and deviant non-target stimuli. N1 windows for children and adults were defined as 150–230 ms for standard non-target and deviant non-target stimuli. Since in both groups a negativity at around 150 ms and a positivity at around 230 ms was also clearly visible at frontal electrode positions these peaks were also assessed. For both the child and adult group, the peak detection window for the first positivity was defined as 120–200 ms for standard and deviant non-target stimuli. The first negativity was defined as 200–260 ms for standard and deviant non-target stimuli.

MMN was computed by subtracting the ERP to the standard non-target stimulus (HFNT) from the ERP to the deviant non-target stimulus (LFNT). Visual inspection of electrode positions O1 and O2 for both group-averaged difference waves and individual subject data indicated that adult subjects displayed a single negative peak around 150 ms post-stimulus, whereas children displayed two negative peaks. In children, the first negativity occurred at around 150 ms and the second one at around 230 ms. Therefore, in the adult group we detected the vMMM as the most negative peak within a 130–200 ms post-stimulus window, while in the child group we detected the first peak as the most negative peak within 130–200 ms post-stimulus, and the second peak as the most negative peak within 200–275 ms post-stimulus. Since a clear positive peak at around 250 ms was also visible in the children's difference wave at frontal (and central) electrode positions, we also assessed this positive peak within 200–275 ms post-stimulus.

### Statistical analyses

All statistical analyses were performed using SPSS 19 (IBM Corp., Armonk, NY, USA). For behavioral analyses, independent samples *T*-tests were performed. For between-groups comparisons of ERP peaks repeated measures mixed model ANOVAs were used, with between subject factor Group (child vs. adult) and within-subject factors Stimulus (standard vs. deviant non-target), and Electrode position (O1 vs. O2; or F3 vs. F4). If Stimulus effects or interactions with Group were significant, follow-up repeated measures ANOVAs were fit for each group separately.

## Results

### Behavioral data

Response accuracy (percentage of correct responses) and reaction times for target conditions are indicated in Table 1. There was a significant difference in the response accuracy between the children and the adults for the deviant background target condition (*t* = 2.38, *p* = 0.022), whereas response accuracy for the standard background target condition did not differ (*p* > 0.08). All individuals across both groups performed the task with at least 95% accuracy. There were no significant differences in mean reaction time between the children and the adults (*p* > 0.1 for both conditions). These results show that both children and adults performed the task with high accuracy and were focusing their attention onto the center of the monitor.

**Table 1:** **Behavioral data for target stimuli in adult (*N* = 20) and child (*N* = 21) groups**.

**Performance**	**Background**	***N***	**Mean**	***SD***
Adult accuracy	Dev	20	100%	0%
Child accuracy	Dev	21	96.90%	5.8%
Adult accuracy	Std	20	98.90%	3.6%
Child accuracy	Std	21	95.48%	5.7%
Adult reaction time	Dev	20	498 ms	50 ms
Child reaction time	Dev	21	590 ms	70 ms
Adult reaction time	Std	20	512 ms	50 ms
Child reaction time	Std	21	598 ms	58 ms

Percentage of correct responses (accuracy) and reaction times in ms [as well as standard deviations (SD)] are indicated for both standard background (Std) and deviant background (Dev) target stimulus conditions.

### Electrophysiological data

**Figure 2:**
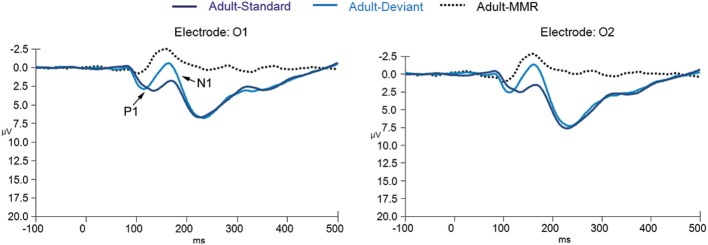
**ERPs for deviant non-target and standard non-target stimulus conditions as well as the difference wave computed by subtracting standard non-target from deviant non-target ERPs in adults (*N* = 20) at electrode positions O1 and O2**.

**Figure 3:**
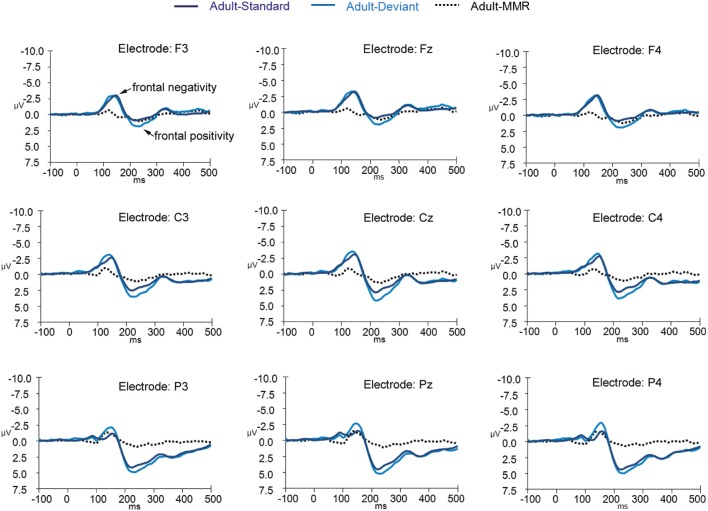
**ERPs for deviant non-target and standard non-target stimulus conditions as well as the difference wave computed by subtracting standard non-target from deviant non-target ERPs in adults (*N* = 20) at electrode positions F3, Fz, F4, C3, Cz, C4, P3, Pz, and P4**.

**Figure 4:**
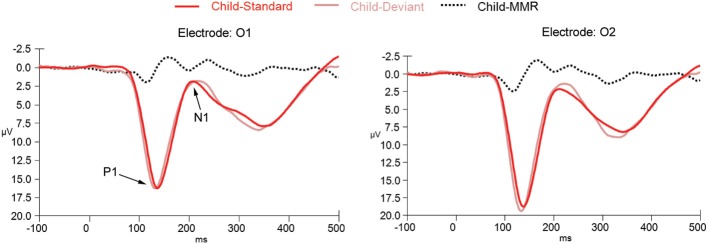
**ERPs for deviant non-target and standard non-target stimulus conditions as well as the difference wave computed by subtracting standard non-target from deviant non-target ERPs in children (*N* = 21) at electrode positions O1 and O2**.

**Figure 5:**
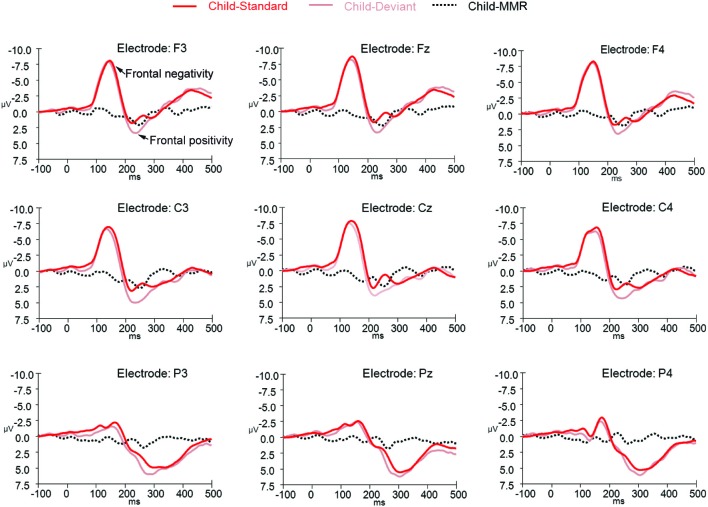
**ERPs for deviant non-target and standard non-target stimulus conditions as well as the difference wave computed by subtracting standard non-target from deviant non-target ERPs in children (*N* = 21) at electrode positions F3, Fz, F4, C3, Cz, C4, P3, Pz, and P4**.

**Figure 6:**
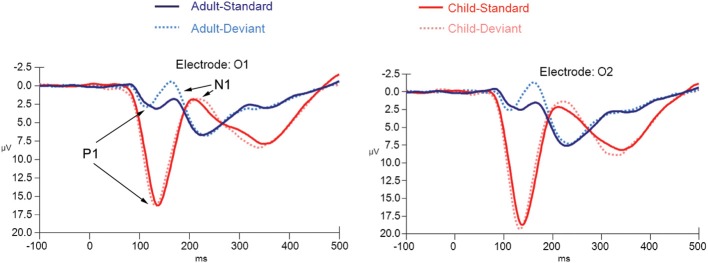
**ERPs for both deviant non-target and standard non-target stimulus conditions in children (*N* = 21) and adults (*N* = 20) at electrode positions O1 and O2**.

**Figure 7:**
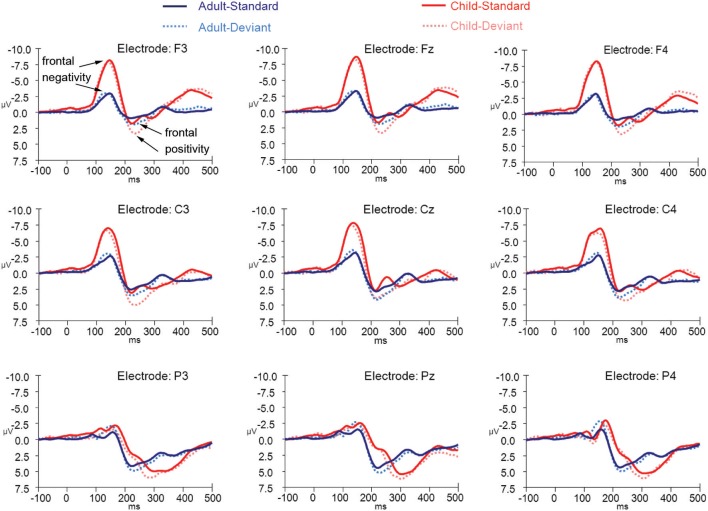
**ERPs for both deviant non-target and standard non-target stimulus conditions in children (*N* = 21) and adults (*N* = 20) at electrode positions F3, Fz, F4, C3, Cz, C4, P3, Pz, and P4**.

ERPs to standard and deviant non-target background stimuli as well as the difference wave (deviant-standard) for adults are shown in Figure 2 (for occipital electrode positions) and Figure 3 (for frontal, central, and parietal electrode positions). For children this is shown in Figures 4, 5. ERPs to standard and deviant non-target background stimuli overlaid for both adults and children are shown in Figure 6 (for occipital electrode positions) and Figure 7 (for frontal, central, and parietal electrode positions). Mean amplitudes and standard deviations are listed in the Appendix (Table A1).

#### P1: amplitude

The repeated measures mixed ANOVA for P1 amplitude at the occipital electrode positions demonstrated a main effect of Group [*F*_(1, 39)_ = 65.49, *p* < 0.001] in the absence of an interaction effect of Stimulus × Group [*F*_(1, 39)_ = 0.37, *p* = 0.684] or an effect of Stimulus [*F*_(1, 39)_ = 0.267, *p* = 0.608], indicating that the P1 amplitude to both standard and deviant non-target stimuli was larger in children than in adults. Furthermore, a significant Electrode position × Group interaction [*F*_(1, 39)_ = 6.34, *p* = 0.016] was observed, indicating that for the children only, the P1 amplitude to standard and deviant non-target stimuli was larger at electrode position O2 than at electrode position O1. No other effects for P1 amplitude were observed.

#### P1: latency

The repeated measures mixed ANOVA for P1 peak latency at occipital electrode positions demonstrated a significant effect of Group [*F*_(1, 39)_ = 16.04, *p* < 0.001] and a significant main effect of Stimulus [*F*_(1, 39)_ = 29.88, *p* < 0.001] in the absence of a Stimulus × Group interaction [*F*_(1, 39)_ = 0.21, *p* = 0.648]. No other effects for P1 peak latency were observed. Since a significant main effect for Stimulus was observed we performed follow-up exploratory within group analyses to test whether the P1 peak latency effect of Stimulus held up for both groups separately.

A significant effect for Stimulus, in the absence of any other effect, was observed for both the adult [*F*_(1, 19)_ = 10.24, *p* = 0.005] and child [*F*_(1, 20)_ = 35.81, *p* < 0.001] group, indicating that for both groups the P1 to deviant stimuli peaked earlier than the P1 to the standard stimuli.

#### Frontal negativity: amplitude

Statistical tests for the negativity occurring at around 150 ms (and for the positivity occurring at around 230 ms) at the fronto-central electrode positions were performed taking only frontal electrode positions into account, since responses were generally largest at those electrode positions. Furthermore, to limit the number of tests and to make comparison to the occipital electrode tests easier to interpret, only electrode positions F3 and F4 were included into the factor “Electrode position.”

The repeated measures mixed ANOVA for the first negativity at around 150 ms at the frontal electrode positions demonstrated a main effect of Group [*F*_(1, 39)_ = 61.19, *p* < 0.001] in the absence of an interaction effect of Stimulus × Group [*F*_(1, 39)_ = 0.006, *p* = 0.937] or an effect of Stimulus [*F*_(1, 39)_ = 1.18, *p* = 0.284]. No other effects were observed. This pattern of results indicates that the amplitude of this peak to both standard and deviant non-target stimuli was larger in children than in adults.

#### Frontal negativity: latency

The repeated measures mixed ANOVA for peak latency of the negativity at around 150 ms at the frontal electrode positions demonstrated no main effect of Group [*F*_(1, 39)_ = 0.19, *p* = 0.684], but did show a main effect of Stimulus [*F*_(1, 39)_ = 7.68, *p* = 0.009] and a main effect of Electrode position [*F*_(1, 39)_ = 9.76, *p* = 0.003]. No other effects were observed. Since a significant main effect of Stimulus was observed we performed follow-up exploratory within group analyses to test whether the effect of Stimulus held up for both groups separately.

In the adult group, a significant effect of Stimulus was observed [*F*_(1, 19)_ = 5.27, *p* = 0.033] in the absence of any other effects, indicating that this negativity peaked earlier in the deviant stimulus condition than in the standard stimulus condition.

In the child group, no significant effect of Stimulus was observed, [*F*_(1, 20)_ = 3.02, *p* = 0.098], but a significant effect of Electrode position [*F*_(1, 20)_ = 8.98, *p* = 0.007] was observed. These results indicate that the latency of the negativity peak did not differ enough between standard and deviant non-target stimuli to reach significance, whereas it did peak earlier at electrode channel F3 than at electrode channel F4.

#### N1: amplitude

The repeated measures mixed ANOVA for N1 amplitude at the occipital electrode positions didn't show a significant effect of Group [*F*_(1, 39)_ = 0.007, *p* = 0.94]. However, a significant main effect of Stimulus [*F*_(1, 39)_ = 9.59, *p* = 0.004] and a trend for a Stimulus × Group interaction [*F*_(1, 39)_ = 3.22, *p* = 0.08] effect was observed. No other effects for N1 amplitude were observed. Since a significant main effect for Stimulus and a trend for a Stimulus × Group interaction were observed, we performed follow-up exploratory within group analyses to test whether the N1 effect of Stimulus held up for both groups separately.

In the adult group, significant effects of Stimulus [*F*_(1, 19)_ = 27.88, *p* < 0.001] and Electrode location [*F*_(1, 39)_ = 8.36, *p* = 0.009] were observed, indicating that the N1 amplitude to deviant non-target stimuli was larger than the amplitude to standard non-target stimuli and that the amplitude on electrode position O2 was larger than the amplitude on electrode position O1.

In the child group, no significant effects were observed, indicating that N1 amplitudes did not differ enough between standard and deviant non-target stimuli and between occipital electrode positions to reach significance.

#### N1: latency

The repeated measures mixed ANOVA for N1 latency at the occipital electrode positions demonstrated a main effect of Group [*F*_(1, 39)_ = 61.80, *p* < 0.001] in the absence of any other effect, indicating that the N1 to both standard and deviant non-target stimuli peaked later in the children than in the adults.

#### Frontal positivity: amplitude

The repeated measures mixed ANOVA for the positivity at around 230 ms at the frontal electrode positions demonstrated a main effect of Group [*F*_(1, 39)_ = 4.12, *p* = 0.049], and significant effect of Stimulus [*F*_(1, 39)_ = 18.77, *p* < 0.001]. No other effects were observed. This pattern of results indicates that the peak amplitude at around 230 ms to both standard and deviant non-target stimuli was larger in children than in adults. Since a significant main effect of Stimulus was observed, we performed follow-up exploratory within group analyses to test whether the effect of Stimulus held up for both groups separately.

In the adult group, a significant effect of Stimulus [*F*_(1, 19)_ = 6.46, *p* = 0.020] was observed, in the absence of any other effects, indicating that the amplitude of the positivity at around 230 ms to deviant non-target stimuli was larger than the amplitude to standard non-target stimuli.

In the child group, a significant effect of Stimulus [*F*_(1, 20)_ = 12.72, *p* = 0.002] was observed, in the absence of any other effects, indicating that the amplitude of the positivity at around 230 ms to deviant non-target stimuli was larger than the amplitude to standard non-target stimuli.

#### Frontal positivity: latency

The repeated measures mixed ANOVA for latency of the positivity at around 230 ms at the frontal electrode positions demonstrated a main effect of Group [*F*_(1, 39)_ = 6.25, *p* = 0.017] in the absence of any other effect, indicating that the positivity at around 230 ms to both standard and deviant non-target stimuli peaked later in the children than in the adults.

#### Difference waves

**Figure 8:**
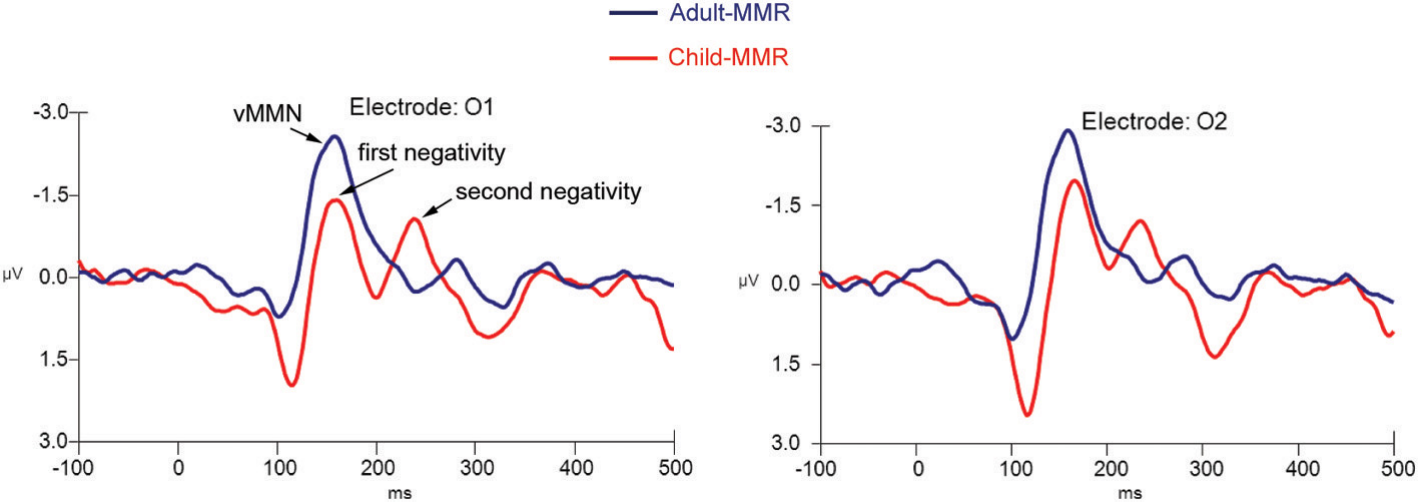
**Difference wave computed by subtracting standard non-target from deviant non-target ERPs for both children (*N* = 21) and adults (*N* = 20) at electrode positions O1 and O2**.

**Figure 9:**
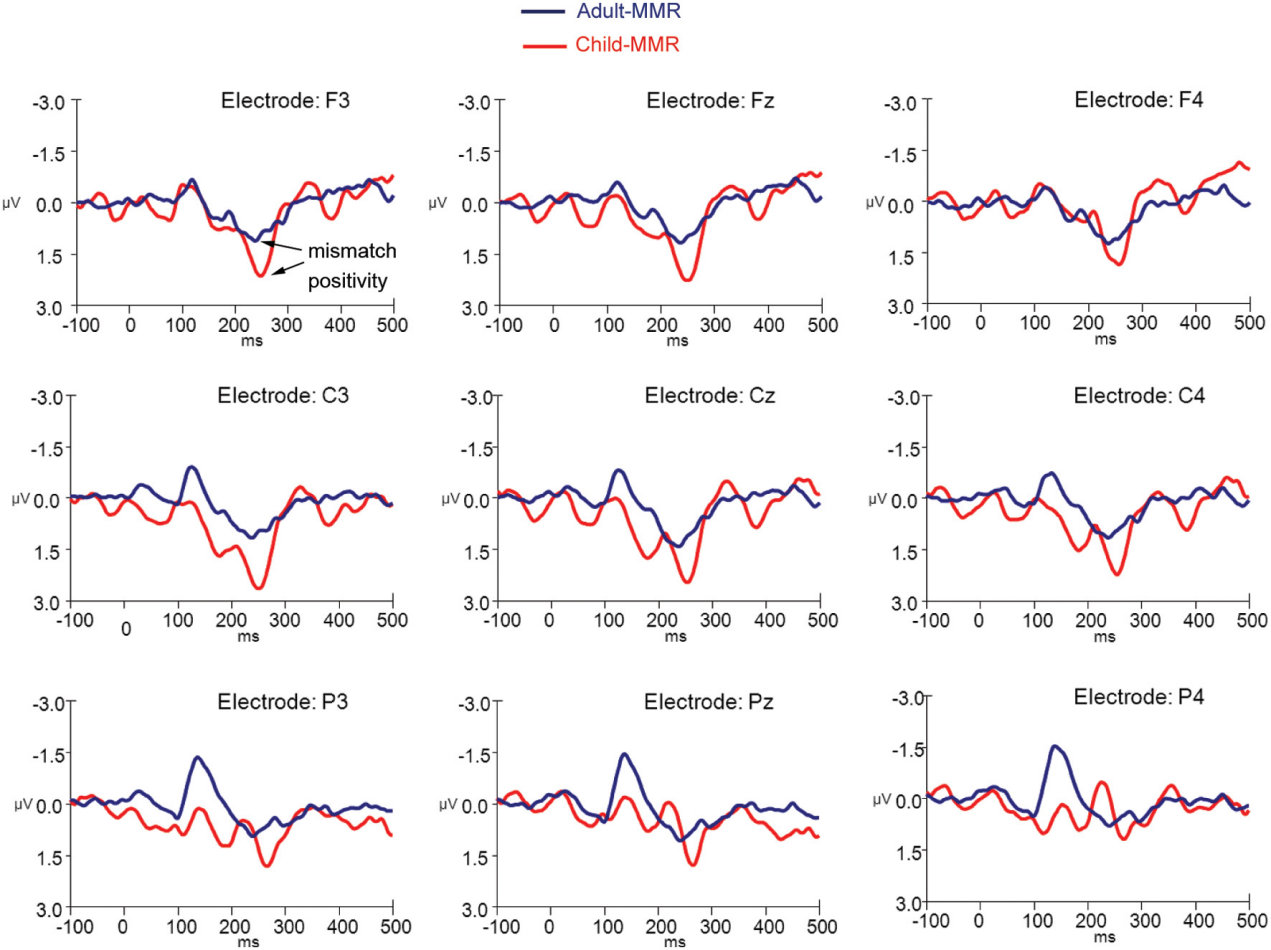
**Difference wave computed by subtracting standard non-target from deviant non-target ERPs for both children (*N* = 21) and adults (*N* = 20) at electrode positions F3, Fz, F4, C3, Cz, C4, P3, Pz, and P4**.

Difference waves (deviant non-target stimuli – standard non-target stimuli) for adults and children are shown in Figure 8 (for occipital electrode positions) and Figure 9 (for frontal, central, and parietal electrode positions).

We first compared the single occipital negativity occurring in the difference wave of the adult group, the two occipital negativities occurring in the difference wave of in the child group and the frontal positivities occurring in the difference waves of both groups against the average amplitude of the baseline period (−200 to 0 ms) to find out whether these peaks significantly differed from “0.” Hereto, a repeated measures ANOVA with factors Stimulus (difference wave response vs. average baseline response), and Electrode position (O1 vs. O2; or F3 vs. F4) was used.

The repeated measures within (adult) group ANOVA for the amplitude of the negativity occurring at around 150 ms against the average baseline activity at occipital electrode positions demonstrated a main effect of Stimulus [*F*_(1, 19)_ = 41.87, *p* < 0.001] in the absence of any other effects.

The repeated measures within (child) group ANOVA for the amplitude of the first negativity against the average baseline activity at occipital electrode positions demonstrated a main effect of Stimulus [*F*_(1, 20)_ = 13.93, *p* = 0.001] in the absence of any other effects. This was also the case for the second negativity {main effect of Stimulus: [*F*_(1, 20)_ = 20.08, *p* < 0.001]}.

The repeated measures within (adult) group ANOVA for the amplitude of the positivity at around 250 ms against the average baseline activity at frontal electrode positions demonstrated a main effect of Stimulus [*F*_(1, 19)_ = 40.85, *p* ≤ 0.001] in the absence of any other effects.

The repeated measures within (child) group ANOVA for the amplitude of the positivity at around 250 ms against the average baseline activity at frontal electrode positions demonstrated a main effect of Stimulus [*F*_(1, 20)_ = 30.55, *p* ≤ 0.001] in the absence of any other effects.

These results indicate that, for both groups, responses apparent in the difference wave over occipital electrode positions as well as over frontal electrode positions significantly differed from the average baseline amplitude.

Finally, we directly compared the difference wave responses that were occurring at around the same point in time between both groups. Hereto, a repeated measures mixed ANOVA with between subject factor Group (child vs. adult) and within subject factor Electrode (O1 vs. O2 or F3 vs. F4) was used.

Although the (first) negative difference wave^1^ occurring at the occipital electrode positions appeared to be larger in the adult group than in the child group, the repeated measures mixed ANOVA indicated that the amplitude difference was not statistically significant [*F*_(1, 39)_ = 5.24, *p* = 0.043] between groups. No other effects for amplitude or latency were observed.

The repeated measures mixed ANOVA for the amplitude of the positivity occurring in the difference wave^1^ at around 250 ms at the frontal electrode positions demonstrated a main effect of Group [*F*_(1, 39)_ = 4.98, *p* = 0.031], in the absence of any other effects, indicating that the amplitude of this positivity at around 250 ms was larger in the child group than in the adult group. No other effects for amplitude or latency were observed.

## Discussion

This study investigated the vMMR in healthy children as compared to healthy adults using a simple visual target detection task, during which task irrelevant gratings of high and low spatial frequencies were presented in the background. We found a robust vMMN in the difference wave (deviant non-target stimuli – standard non-target stimuli) occurring around 150 post-stimulus over occipital electrode positions in the adult group, and two occipital negativities in the children, the first one occurring at around 150 ms and a second one at around 230 ms. We also observed a positivity at frontal and central electrode positions at around 250 ms in both groups. This study confirms previous research investigating vMMN in healthy adults and is one of the first to investigate this difference wave in children aged 8–12 years old. The results indicate that both children and adults respond to the occurrence of rare task irrelevant visually deviant stimuli, although this response is still developing in healthy children ages eight to twelve and may be quite different in this age group in terms of morphology (amplitude, latency) and topography (occipital negativities, fronto-central positivity) compared to typically-developing adults.

Our results differ from previous work by Clery et al. (2012), in which changes in form and motion resulted in three sequential negative and one positive response in 8–14 year old children while only one negative response was observed in adults. We observed two negativities and one positivity in the difference waves in our study. Clery et al. argue that multiple peaks may be due to a sequential visual processing of deviancy necessary in the developing brain but not in the mature brain. Our results generally support this hypothesis, however, the inconsistent findings concerning number of negativities may indicate that these peaks are more dependent on individual differences, or are undergoing developmental changes in this age range. The differences between our results and those of Clery et al. (2012) could also be due to the different nature of the stimuli used and the properties each investigates: Clery et al. point out that it is difficult to determine whether their results were driven by changes in form, motion, or both. Perhaps less dynamic stimuli such as the ones used in our study impose reduced processing demands, insufficient to activate the third waveform observed by Clery et al. It would be interesting to determine if multiple peaks can be elicited with static stimuli of increasing complexity, or if this is due to the dynamism of a stimulus alone.

A limitation of this study is that it could be argued that stimulus effects from the use of low frequency gratings as deviant stimuli may account for the vMMN seen here. Spatial frequency deviance has been previously studied by Heslenfeld (2003), where differences in ERPs were indeed observed based on different spatial frequencies. Some behavioral differences were also observed: e.g., task-irrelevant stimuli of low spatial frequencies were more likely to interfere with performance than HSF stimuli, but only in difficult tasks. However, our task was not demanding and all subjects performed it easily and accurately, including the youngest children. In the previous study by Heslenfeld (2003), ERP effects were observed in different components of the ERP and different electrode sites than are studied here, such as a larger early C1 component (60–100 ms) in HSF gratings vs. low, as well as larger responses at frontal and central scalp sites at 120–180 ms in LSF stimuli vs. high. Heslenfeld concluded that this deviance was due to stimulus effects and was congruent with previous literature, which found higher response-interference and attention-capturing properties of low spatial frequencies. However, the effects at occipital sites (120–200 ms) were independent of task load or spatial frequency, showing that this response was not related to individual stimulus properties or refractoriness. Hence, this negativity is likely the true visual analog of the auditory MMN because it is not related to stimulus features or task difficulty. Our results in the adult group show a negativity at comparable electrode locations and latency. Similar effects have been observed in other studies using the equiprobable paradigm (Czigler et al., 2006; Kimura et al., 2009), where two negativities were found but only one was attributed to stimulus-independent visual deviance. We believe that the mismatch effects observed in the current study are not solely related to refractoriness or spatial frequency effects although our study design did not allow for excluding this possibility. In the child group, two occipital negativities were observed. The second occipital peak co-occurs with the frontal positivity observed at around 250 ms. This may suggest recruitment of higher-order cognitive processes with a more frontally located brain source. However, more research is needed to confirm this hypothesis. We should also point out the fact that we examined the process of automatic visual deviance detection while participants were engaged in a visual target detection task. Hence all task stimuli were presented in the same modality. However, in a typical auditory MMN paradigm the participants' attention is usually directed toward another (e.g., visual) modality. Participants are asked to read a book or watch a movie for instance. Keeping attention focused within the same modality as opposed to dividing attention between the auditory and the visual domain may differentially impact the vMMR. Future studies could examine the possible effect of this on the vMMN.

There were also differences in other ERP components between adults and children: as seen previously in the literature, early components, particularly the amplitude of the P1 was larger in children and both the P1 and N1 peaked later in children. Batty and Taylor (2002) also noted this effect in a simple visual categorization task, finding that the amplitude of P1 seemed to decrease with age throughout adolescence. In our study, amplitude of the P1 was also larger and the peak more broad, resulting in a much later N1 in children vs. adults. It could be that underlying neural mechanisms are underdeveloped in children and/or that they may employ fewer response strategies when performing this particular task (i.e., concerns about speed, accuracy, and impulsivity management, and attention devoted to the task's purpose). Behavioral reports on subjects' experience of the task following the ERP experiment might help to answer this question.

This study adds to the limited pool of studies investigating vMMR in children. Due to the preliminary nature of this study, and aware of the developing cognitive system and accompanying changes in ERPs that tend to occur across the lifespan, we chose a limited age range to determine initial differences between children and adults. However, future research should examine other and even narrower age ranges in order to better map the development of vMMR. Our stimuli also probed only one aspect of automatic visual deviancy detection (spatial frequency), and future work should investigate other stimulus properties such as color, luminance, and size, to further understand development of the visual deviance response.

Considerations for future studies should also include investigating abnormal development of vMMR. Individuals with schizophrenia have been found to exhibit reduced amplitudes of vMMN when compared to healthy controls (Urban et al., 2008). Furthermore, reduced vMMN amplitude was found to be associated with lower levels of functioning in schizophrenia, as well as with higher levels of medication dosage. In another study, Qiu et al. (2011) found decreased vMMN amplitudes in individuals with major depressive disorder, although this difference did not correlate with depression severity.

Although the above research has demonstrated the usefulness of vMMN as a potential clinical tool, few studies have investigated altered vMMR in disorders affecting children. To our knowledge there have only been two other studies of vMMR in children with neurodevelopmental disorders (Horimoto et al., 2002; Clery et al., 2013). Visual MMR could be useful to probe visual information processing deficits in children with neurodevelopmental disabilities, and future work should investigate what differences in vMMR, if any, might occur in atypical neurodevelopment.

### Conflict of interest statement

The authors declare that the research was conducted in the absence of any commercial or financial relationships that could be construed as a potential conflict of interest.

## Footnotes

^1^Note that the cognitive process underlying this difference wave response may not be identical in adults and children.

## Appendix

**Table A1:** **Peak amplitude values and latencies for ERPs and difference waves for electrode positions O1, O2, F3, and F4**.

**Peak**	**Channel**							
	**O1**		**O2**		**F3**		**F4**	
	**Amp**	**Lat**	**Amp**	**Lat**	**Amp**	**Lat**	**Amp**	**Lat**
**ADULT GROUP**								
P1 Std	4.3(2.9)^+^	126(16)^+^	3.9(3.3)^+^	123(17)^+^	n/a	n/a	n/a	n/a
P1 Dev	4.3(2.4)^+^	119(16)^+^	4.0(2.8)^+^	117(18)^+^	n/a	n/a	n/a	n/a
N1 Std	0.6(2.3)	165(18)^+^	−0.5(2.9)	166(20)^+^	n/a	n/a	n/a	n/a
N1 Dev	−1.5(3.0)	167(23)^+^	−2.5(3.4)	166(20)^+^	n/a	n/a	n/a	n/a
Frontal Neg Std	n/a	n/a	n/a	n/a	3.3(1.7)^+^	144(15)	3.4(1.8)^+^	146(13)
Frontal Neg Dev	n/a	n/a	n/a	n/a	3.7(2.2)^+^	139(19)	3.6(2.0)^+^	141(15)
Frontal Pos Std	n/a	n/a	n/a	n/a	1.5(2.2)^+^	216(22)^+^	1.5(2.3)^+^	218(22)^+^
Frontal Pos Dev	n/a	n/a	n/a	n/a	2.4(2.6)^+^	223(17)^+^	2.5(2.8)^+^	224(15)^+^
**DIFFWAVE**								
Occipital MMN	−3.1(2.0)^*^	162(20)	−2.7(2.6)^*^	156(16)	n/a	n/a	n/a	n/a
Frontal Pos	n/a	n/a	n/a	n/a	1.6(1.2)^+^^*^	229(17)	1.6(1.2)^+^^*^	226(18)
**CHILD GROUP**								
P1 Std	16.7(6.0)	138(7)	19.5(7.8)	138(6)	n/a	n/a	n/a	n/a
P1 Dev	17.0(8.2)	132(7)	20.0(9.6)	133(7)	n/a	n/a	n/a	n/a
N1 Std	−0.8(5.2)	224(31)	−0.3(6.1)	221(26)	n/a	n/a	n/a	n/a
N1 Dev	−1.1(6.2)	223(30)	−1.2(6.3)	223(31)	n/a	n/a	n/a	n/a
Frontal Neg Std	n/a	n/a	n/a	n/a	8.5(2.3)	145(12)	8.8(2.7)	149(11)
Frontal Neg Dev	n/a	n/a	n/a	n/a	8.7(2.6)	140(12)	9.1(2.8)	144(15)
Frontal Pos Std	n/a	n/a	n/a	n/a	3.0(4.0)	232(23)	3.0(3.8)	236(23)
Frontal Pos Dev	n/a	n/a	n/a	n/a	5.2(4.5)	233(27)	4.8(4.2)	234(25)
**DIFFWAVE**								
Occipital 1st Neg	−2.9(3.6)^*^	155(13)	−2.6(3.0)^*^	157(12)	n/a	n/a	n/a	n/a
Occipital 2nd Neg	−2.1(3.2)^*^	228(26)	−2.5(3.0)^*^	233(30)	n/a	n/a	n/a	n/a
Frontal Pos	n/a	n/a	n/a	n/a	2.9(2.9)^*^	229(31)	3.1(3.1)^*^	230(32)

ERP peak amplitude values and latencies as well as their respective standard deviations (in parentheses) for standard non-target (Std) and deviant nontarget (Dev) background stimulus conditions in adult (N = 20) and child (N = 21) groups. Neg, Negativity; Pos, Positivity; Diffwave, Difference wave; MMN, Mismatch Negativity;

+ , *significantly different from child group with p < 0.05;*

* , *significantly differs from average baseline activity with p < 0.05 (within group); n/a, non-applicable.*
